# Mortalin promotes the evolution of androgen-independent prostate cancer through Wnt/β-catenin signaling pathway

**DOI:** 10.1186/s12935-024-03345-x

**Published:** 2024-06-07

**Authors:** Ying Chang, Jinyuan Sui, Qiang Fu, Zhongqi Lu, Zhengri Piao, Tiefeng Jin, Meihua Zhang

**Affiliations:** 1 Department of Health Examination Centre, Affiliated Yanbian University Hospital, Yanji, China; 2 Department of Ultrasound Medicine, Affiliated Yanbian University Hospital, Yanji, 133000 China; 3 Department of Pathology and Cancer Research Center, Yanbian University Medical College, Gong Yuan Road No.977, Yanji, 133000 China; 4 Key Laboratory of the Science and Technology, Department of Jilin Province, Yanji, China; 5 Department of Radiology, Affiliated Yanbian University Hospital, Yanji, 133000 China

**Keywords:** Mortalin, Androgen-independent prostate cancer, Wnt/β-catenin, Therapeutic target

## Abstract

Prostate cancer (PC) is a major global health concern affecting male individuals. Among its variants, androgen-independent prostate cancer exhibits slow progression and lacks effective treatment targets, rendering it insensitive to hormone therapy. Recent reports have highlighted the significance of Mortalin, an important oncogene, in tumor migration and invasion through various signaling pathways. Experimental evidence from in-vivo and in-vitro studies indicate upregulated expression of Mortalin in prostate cancer tissues. Moreover, it has been shown to regulate the epithelial-mesenchymal transition (EMT) process via the Wnt/β-catenin signaling pathway, thereby promoting prostate cancer proliferation and metastasis. These findings suggest that Mortalin may serve as a promising novel immunotherapeutic target for prostate cancer.

**Supplementary Information:**

The online version contains supplementary material available at 10.1186/s12935-024-03345-x.

## Introduction

Cancer and other non-communicable diseases (NCDs) are now widely recognized as a threat to global development, an observation reiterated by the latest United Nations High-level Meeting on NCDs [1, 2]. The aging population is leading to an increased cancer burden in older people. The latest global cancer data for 2023 show that prostate cancer (PC) is a significant global health concern, particularly among the aging population. As the latest data highlight, PC remains one of the most prevalent cancers among men, with a high rate of new morbidity and mortality [3]. In men on active surveillance older age at diagnosis was positively associated that imply for many older men, active surveillance as opposed to watchful waiting remains a more appropriate management strategy [4]. Androgen-dependent prostate cancer, also known as hormone-sensitive prostate cancer, has indeed been the focus of treatment with a variety of therapies targeting the androgen receptor signaling pathway. Drugs like flutamide and apalutamide are examples of androgen receptor inhibitors commonly used in the clinic for treating this type of prostate cancer [5]. Androgen-independent prostate cancer is difficult to treat once diagnosed because of its slow progression, lack of effective therapeutic targets and insensitivity to hormonal therapy. Thus, it is very important to find effective treatment, immunotherapy is currently a hotspot in the treatment of cancer [6]. Localized diseases can be successfully treated, but advanced cases have more problems [7]. The fact that early-stage prostate cancer is usually asymptomatic emphasizes the need for research and development of new diagnostic and treatment methods, as well as the use of combination therapy to improve treatment efficacy. This is because the disease is often diagnosed in advanced or even metastatic stages, making it difficult to treat. Therefore, it is crucial to develop new diagnostic methods and treatment options to improve outcomes for patients with prostate cancer [8].

Mortalin, also known as GRP75/HSPA9, is a molecular chaperone of mitochondrial motility related proteins in the HSP70 family, is a highly conserved mitochondrial chaperone protein. It is involved in many cellular activities, including protein folding and transport, maintenance of mitochondrial stability, substance synthesis [9, 10], apoptosis, senescence and intracellular signaling pathways [11, 12]. Mortalin is associated with tumor metastasis and highly expressed in a variety of human tumors [13]. Recent studies have revealed the involvement of Mortalin in cancer progression, highlighting its potential as a therapeutic target in several malignancies [14, 15]. Mortalin levels in tumor tissues are higher than those in healthy tissues and associated with the proliferation of cancer cells, activating the Wnt/β-catenin pathway to accelerate epithelial-mesenchymal transition (EMT) [16, 17]. EMT is closely associated with tumor metastasis, which acquires the ability to migrate and invade due to loss of cell membrane polarity and reduced adhesion [18]. Mortalin may be involved in the EMT process, but the exact mechanism of Mortalin’s role in prostate cancer remains unclear [13, 19]. Co-activation of ALK and N-myc can induce androgen-independent prostate cancer by stimulating the Wnt/β-catenin pathway, which can be used for targeted tumor therapy [20]. It has been improved that Mortalin plays a vital role in the progression of prostate cancer, but the mechanism of its association with androgen-independent prostate cancer is still unknown.

We investigated the impact of Mortalin expression on the progression of prostate cancer through in-vitro and in-vivo experiments and clinical data. Mortalin may play a critical role in the progression of prostate cancer through the Wnt/β-catenin signaling pathway and has the potential to become a therapeutic target for androgen-independent prostate cancer. The upregulation of Mortalin expression in prostate cancer tissues and its promotion of tumor progression through the Wnt/β-catenin signaling pathway suggests the feasibility and efficacy of exploring Mortalin as a targeted therapy for prostate cancer.

## Materials and methods

### Cell lines

The cell lines used, LNCAP, 22RV1, DU145, and PC3, were provided by the Tumor Research Center of Yanbian University. Two independent androgenic cell lines, DU145 and PC3, were selected from prostate cancer cell lines with high malignancy and high expression of Mortalin by database and Western blotting assay. Both cells were cultured in Dulbecco's modified Eagle medium supplemented with 10% FBS, 1% penicillin/streptomycin, and in 37 °C, 5% CO_2_ in a humidified incubator.

### Immunohistochemistry (IHC) staining

The tissue microarray was manufactured by Shanghai Outdo Biotechnology Corporation (Shanghai, China). Clinicopathological data of the samples were shown that 42 cases were under 60 years of age and 48 cases were over 60 years of age. All cases were pathologically confirmed to be in accordance with clinical pathologic diagnostic criteria of prostate cancer.

According to the Gleason score, there were 36 cases with Gleason ≤ 6 and 54 cases with Gleason ≥ 7. According to the TNM stage, there were 57 cases in stage I–II and 33 cases in stage III–IV. IHC staining scores were assessed by two pathologists who had no knowledge of the patient's clinicopathological data. The IHC staining scores used were ‘0’ (negative, −), ‘1–3’ (weak, +), ‘4–6’ (moderate, ++) and “8–12” (strong, +++).

Immunohistochemistry staining was performed on all tissues using the DAKO LSAB kit (DAKO A/S, Glostrup, Denmark). The tissue microarrays were baked at 60 °C, dewaxed with xylene, hydrated through a gradient concentration and repaired by sodium citrate buffer, then incubated at 3% H_2_O_2_ for 20 min. The tissue sections were incubated with Mortalin, Ki-67, C-myc, Cyclin-D1 antibody (1:100, Santa Cruz Biotechnology) overnight at 4 °C, followed by exposure to secondary antibody. Finally, the sections were visualized by 3, 3-diaminobenzidine (DAB) solution and counterstained with hematoxylin.

### Database analysis

Application of cancer cells is an encyclopedia (CCLE) database (https://portals.broadinstitute.org/ccle) to extract the prostate tumor tissues Mortalin mRNA expression. Prostate carcinoma tissues and normal Mortalin expression in tumor tissue analysis of the differences between using UALCA database (http://ualcan.path.uab.edu). The relationship between Wnt/β-catenin pathway and EMT progression was examined using STRING (https://string-db.org), and repeated validation was performed using gene-Mania database (https://genemania.org). The association between Mortalin and Wnt/β-catenin pathway associated proteins DVL2, CTNNB1 and CCND1 in prostate cancer was searched using the GEPIA database (http://gepia.cancer-pku.cn).

### Western blot

In brief, polyacrylamide gel electrophoresis was used to separate proteins which extracted from tissues or cells, and transferred onto polyvinylidene fluoride (PVDF) membranes. Membranes were incubated with primary antibodies including: Mortalin(1:1000; Santa Cruz Biotechnology), β-Actin(1:1000; Proteintech), E-cadherin(1:1000; Proteintech), Vimentin(1:1,000; Santa Cruz Biotechnology), Slug(1:1000; Santa Cruz Biotechnology), Twist(1:1,000; Santa Cruz Biotechnology), VEGF (1:1000; Santa Cruz Biotechnology), MMP2 (1:1000; Santa Cruz Biotechnology), MMP9 (1:1000; Santa Cruz Biotechnology), β-catenin(1:1000; Santa Cruz Biotechnology), C-myc(1:1000; Santa Cruz Biotechnology), Cyclin1-D1(1:1000; Santa Cruz Biotechnology), and with secondary antibody, then quantified by gel imaging system (Bio-RAD, Hercules, CA, USA) and photographed.

### Transfection

A Mortalin-specific small interfering RNA (siRNA) transfection kit, purchased from RiboBiow (Guangzhou, China), was used to knock down the expression of Mortalin in cells. siRNA1 and siRNA3 sequences were GCGATATGATGATCCTGAA and GCTGGAATGGCCTTAGTCA, respectively. Non-specific siRNA was used as negative control (si-con). The mixture containing 1 × buffer (12 μl/well), siRNA (5 μl/well) and transfection buffer (120 μl/well) was configured and added into DMEM without penicillin/streptomycin, then placed in an incubator for 48 h.

### Immunofluorescence (IF)

For immunostaining, cells were cultured on (24 × 24 mm) slides, washed with PBS, fixed with 2% paraformaldehyde for 30 min, permeabilized with 0.5% TritonX-100 (CWBIO, Beijing, China), sealed with BSA (Solarbio, Beijing, China). Primary antibodies specific for the following proteins were used: Mortalin(1:100; Santa Cruz Biotechnology), β-Actin(1:100; Proteintech), E‑cadherin (1:100; Proteintech.), Vimentin (1:100; Santa Cruz Biotechnology), C-myc(1:100; Santa Cruz Biotechnology), Cyclin1-D1(1:100; Santa Cruz Biotechnology). Following incubation with the primary antibodies, the cells were then incubated with Alexa Fluor 488 goat anti‑rabbit IgG (1:400; cat. no. A11008; Invitrogen; Thermo Fisher Scientific, Inc.) or Alexa Fluor 568 goat anti‑mouse IgG (1:400; cat. no. A11004; Invitrogen; Thermo Fisher Scientific, Inc.) at room temperature for 1 h. Sections were sealed with an anti-fading fluorescent fixator containing 4ʹ,6-diamino-2-phenylindole (DAPI, Solarbio). The intensity of fluorescence image was observed and photographed under a microscope.

### MTT assay

After cell digestion and counting, DU145 (5 × 10^3^ cells/well) and PC3 (5 × 10^3^ cells/well) were inoculated on 96-well plates and incubated in an incubator for 0, 24, 48 and 72 h. Then cells were incubated in100μl/well medium with 20 μl (5 mg/l) MTT for 4 h. Replaced the medium with 100 μl dimethylsulfoxide (DMSO). Finally, the optical density (OD) was measured at 490 nm.

### Colony formation assay

PC3 and DU145 cells transfected with Mortalin-specific siRNA or non-specific siRNA were plated at a density was 1000 cells/well, placed in 6-well plates and cultured for at least 14 days. Then the cells were fixed with 100% methanol for 10 min and washed in PBS and stained with 0.5% hematoxylin (Solarbio). Visible colonies in the wells were counted under a light microscope directly.

### Wound healing assay

Cells were incubated at a density of 5 × 10^5^ cells /well in a 6-well plate until density reached nearly 80–90% confluence. The cell was scratched by 200 μl pipette tips. Microscopic photographs of the wound area at 0, 24, 48 and 72 h and analyzed by ImageJ software. Prism 8.0 was used for data statistics.

### Cell migration assay

Transwell migration assay was performed in a 24-well plate. Cells were inoculated at a density of 5 × 10^4^ cells into 0.1 ml serum-free conditioned medium in the upper chamber BD (BD Biosciences, Piscataway, NJ, USA), and 1 ml medium containing 10% FBS was added into the lower chamber. After incubation at 37 °C with 5% CO_2_ for 24 h, paraformaldehyde was fixed, hematoxylin was stained, observed under microscope and photographed.

### Endothelial cell tube formation assay

A 96-well plate was filled with 30 μl of Matrigel (BD Bioscience) and 30 μl of serum-free DMEM without dual antibodies, then incubated in a 37 °C, 5% CO_2_ incubator for 4 h. Cells treated under different conditions (1.5 × 10^3^ cells/well/100 μl) were inoculated vertically on top of the gel and incubated for 6 h in an incubator at 37 °C, 5% CO_2_. Photographs were taken by confocal laser microscopy.

### Animal model

Ten male BALB/c mice aged 5 weeks and weighing approximately 20 g, were procured from Beijing Vital River Laboratory Animal Technology Co, Ltd. Mice were raised in a pathogen free environment at 22 °C, with 50% humidity and a light/dark cycle of 12 h. 1 × 10^6^ PC-3 cells were mixed with Matrigel (BD Biosciences, Franklin Lakes, NJ, USA) and injected intraperitoneally into the right fat pad of the 4th mammary gland of nude mice. The tumor volume was measured using an improved ellipsoidal volume formula (volume = 1/2[length × width^2^]). The Institutional of Yanbian University monitored and approved Animal Ethics (approval no. YD20231027003), and was performed according to the guidelines of the Committee on Animal Research and Ethics.

### US imaging

The A Vevo2100 LAZR high frequency ultrasound (US) imaging system, manufactured by FUJIFILM VisualSonics Inc. in Toronto, Ontario, Canada, was utilized for all US image acquisition. This system is equipped with a linear array transducer (LZ-550), which has a center frequency of 32–55 MHz and an integrated light source. The system utilizes fiber-optic transducers to deliver nanosecond laser pulses into deep anatomical targets. The laser light is differentially and specifically absorbed by tissues, causing transient thermoelastic expansions that generate acoustic pressure waves. These waves are detected by 256 sensitive piezoelectric elements, and the transmitted US pulses are similarly received, generating high-resolution images of microscopic anatomical structures. The photoacoustic and spatial dimensions of the collected US images were less than 14 mm in width and less than 15 mm in depth.

### Statistical analyses

Data are presented as mean ± standard deviation, and used for data statistics and analysis by GraphPad Prism 8.0 software (GraphPad, La Jolla, CA, USA). Database data and clinical patient data were analyzed using one-way AOVA or T-test. *P < 0.05 was considered statistically significant. According to the analysis of experimental results, significance was *P < 0.05, **P < 0.01, ***P < 0.001, or ****P < 0.0001. All experiments were repeated three times.

## Results

### Mortalin protein expression was up-regulated in prostate cancer and positively correlated with the malignancy

**Fig. 1:**
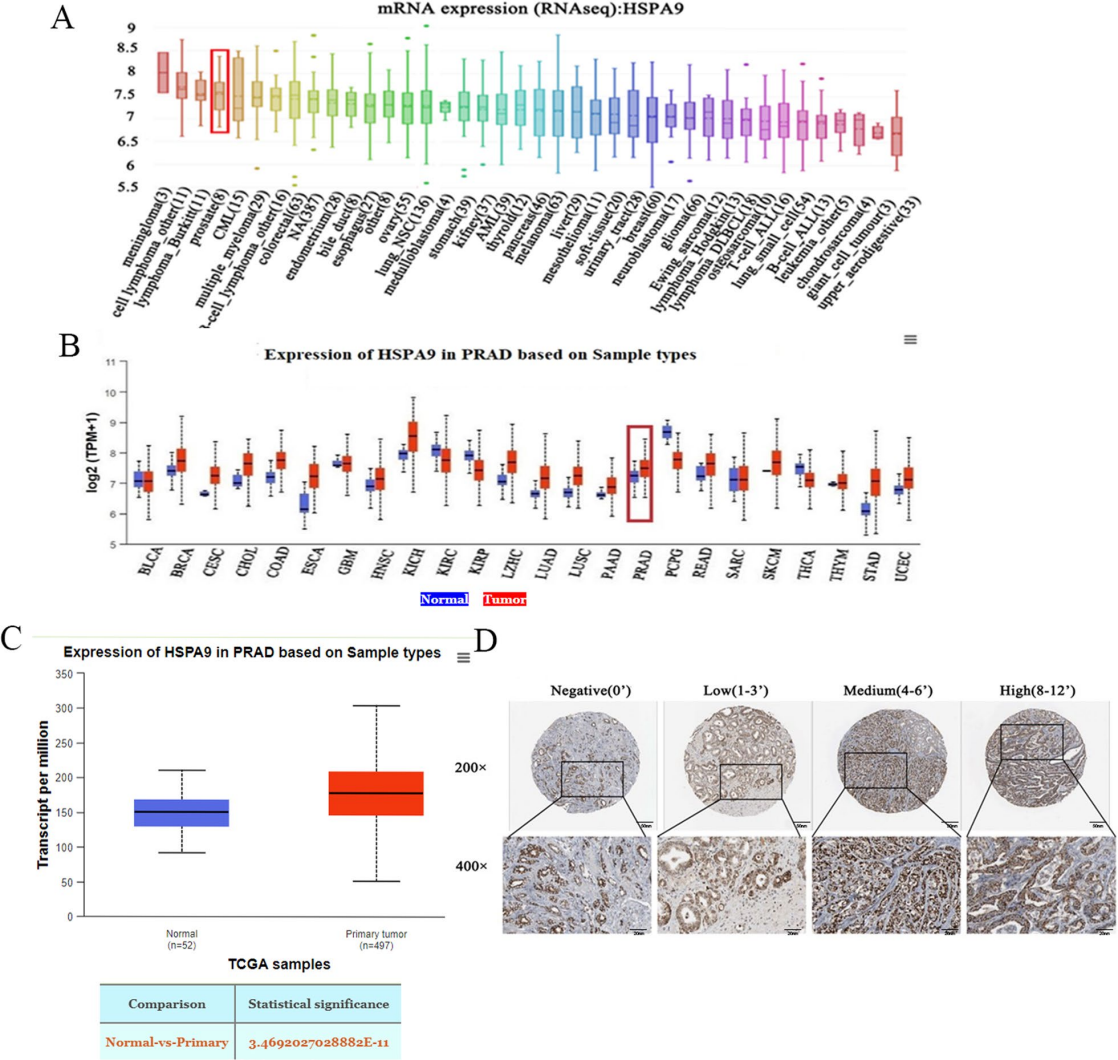
Mortalin expression in prostate cancer. **A** Mortalin mRNA expression in prostate cancer. **B** Mortalin is difference expressed in prostate cancer and matched peritumoral specimens. **C** Differential expression of Mortalin in normal prostate tissues and prostate cancer tissues. **D** Mortalin expression in prostate cancer tissues

To explore the role of Mortalin in cancer development, we analyzed the relative mRNA expression of Mortalin in tumour tissues and adjacent non-tumour tissues through the CCLE database and The Cancer Genome Atlas (TCGA) database, and the results showed that Mortalin was highly expressed in prostate, breast and lung cancers (Fig. 1A–C). Meanwhile, to evaluate whether Mortalin can be used as a prognostic marker for prostate cancer. The intensity of Mortalin expression in prostate cancer tissues was precisely determined using IHC, which examined Mortalin expression in 90 patients with different stages of prostate cancer. The results revealed that Mortalin protein levels were significantly increased in tumor tissues compared to adjacent non-tumor tissues (Fig. 1D). In addition, Mortalin expression was positively correlated with the malignancy of the tumor. According to Gleason score, Mortalin expression was analyzed in 90 patients. The positive rate of Gleason score ≤ 6 was 66.3%, and the strong positive rate was 30.6%. The positive rate of Gleason score ≥ 7 was 83.3% and the strong positive rate was 55.6%. The positive rate of Tumor-surrounding tissue was 31.1%, and the strong positive rate was only 0% (Table 1, Additional file 1A). The higher Gleason score, the higher Mortalin expression, which is consistent with the IHC. The above results indicated that Mortalin is highly expressed in prostate cancer patients.

**Table 1:** Expression of mortalin in prostate cancer tissue microarray

Diagnosis	No	Positive cases				Positive (%)	Strongly positive (%)
		−	+	++	+++		
Cancer Gleason ≥ 7	54	9	6	9	30	83.3	55.6
Cancer Gleason ≤ 6	36	12	8	5	11	66.7	30.6
Adjacent	90	62	26	2	0	31.1	0

### Mortalin promotes proliferation in prostate cancer cells

**Fig. 2:**
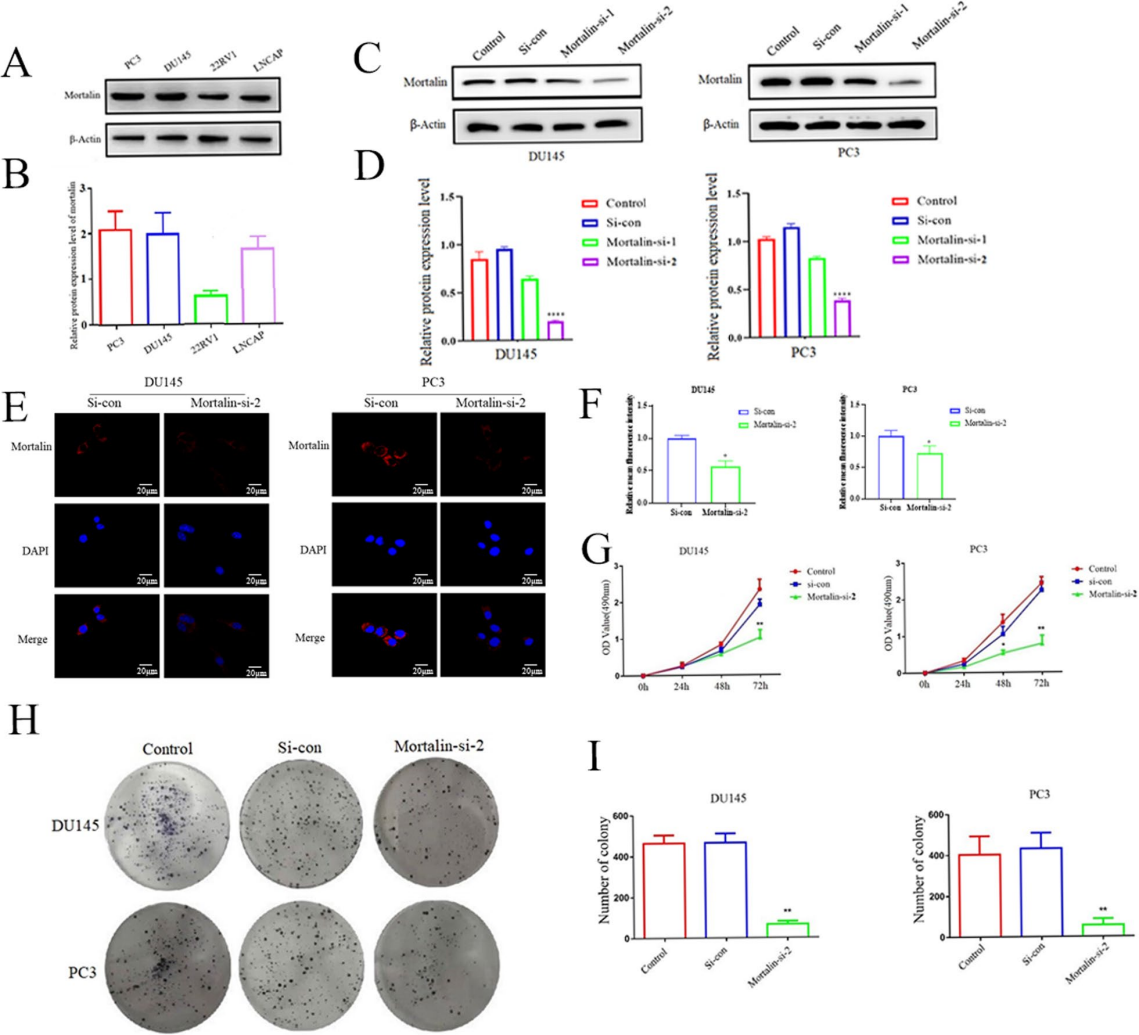
Mortalin expression is related to the proliferation of prostate cancer. **A** Mortalin expression in different prostate cancer cell lines. **B** Statistical histogram of group A data. **C** Western blot was used to detect protein expression in different Mortalin expression groups in PC3 and DU145 cells. **D** Statistical histogram of group C data. **E** Differences in Mortalin expression between DU145 and PC3 cells were detected by immunofluorescence. **F** Statistical histogram of group E data. **G** The growth differences of PC3 and DU145 cells were determined by MTT assay. **H** The ability of colony forming in PC3 and DU145 cells. **I** Statistical histogram of group G data. *P<0.05, **P<0.01, ****P < 0.0001 VS Control

PC3, DU145 and 22RV1 are cell lines for androgen-independent prostate cancer, while LNCAP is the cell line for androgen-dependent prostate cancer, and we used Western blot to detect Mortalin expression in these four cell lines (Fig. 2A, B) and found that Mortalin was expressed higher in PC3 and DU145 cells. In subsequent experiments, Mortalin-specific siRNA was used to knock down the expression of Mortalin in PC3 and DU145 cells, and the expression of Mortalin was detected by western blot (Fig. 2C, D), and the more specific Mortalin-specific siRNA sequence 2 (Mortalin-si-2) was selected for subsequent experiments. At the same time, the protein expression after knocking down Mortalin was repeatedly verified by IF staining, and the results showed that Mortalin expression was significantly reduced after application of Mortalin-si-2 in PC3 and DU145 cells (Fig. 2E). In this paper, MTT method and colony formation method were used to detect whether knockdown Mortalin protein can inhibit cell proliferation of PC3 and DU145, and the results showed that knockdown Mortalin can significantly inhibit the proliferation and clonal formation ability of prostate cancer cells compared with the control group (Fig. 2F–H). In conclusion, down-regulating Mortalin expression inhibits the proliferation of prostate cancer cells.

### Mortalin promotes metastasis and angiogenesis of prostate cancer

**Fig. 3:**
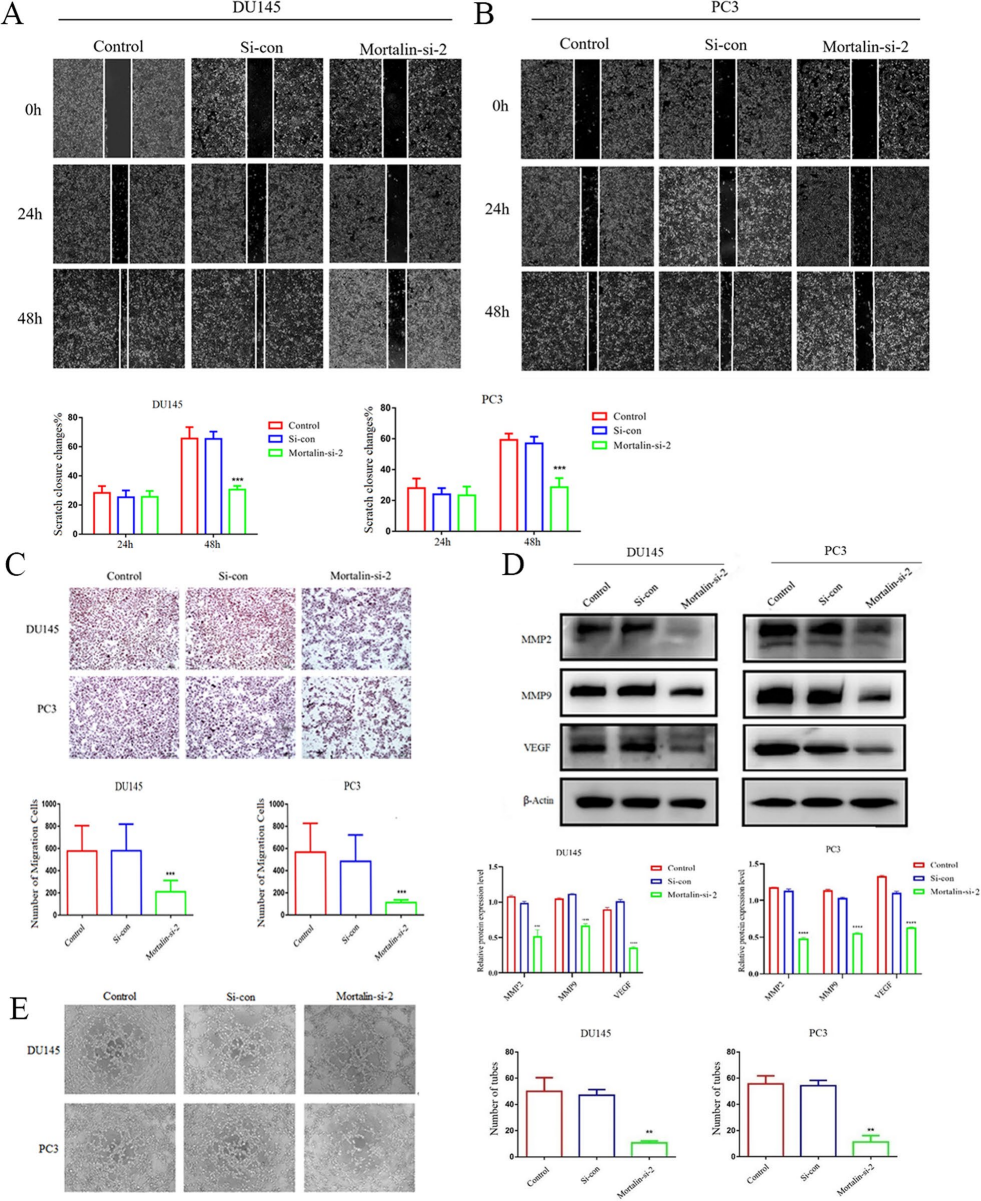
Mortalin promotes the migration in prostate cancer cells. **A** Effect on wound healing ability in DU145 cells with differential expression of Mortalin. **B** Effect on wound healing ability in PC3 cells with differential expression of Mortalin. **C** Effect on migration ability of PC3 and DU145 cells with differential expression of Mortalin. **D** Mortalin down-regulation influences expression of MMP2, MMP9 and VEGF, and western blotting histogram. **E** Effect on angiogenesis ability with differential expression of Mortalin, and angiogenesis ability histogram. **P < 0.01, ***P<0.001, ****P < 0.0001VS Control

Tumor metastasis is a major cause of death in cancer patients and inhibition of tumor migration is important in tumor therapy. We used the wound healing assay and Transwell assay to examine the effects of knocking down Mortalin expression on migration and invasion, respectively. The results showed that knockdown of Mortalin expression could effectively inhibit the wound healing ability of prostate cancer cells and inhibit cell migration (Fig. 3A, B). At the same time, reducing Mortalin expression inhibited the invasive effect of tumor (Fig. 3C). Western blot results also demonstrated that knockdown of Mortalin inhibited the expression of MMP2 and MMP9, proteins involved in tumor progression and metastasis (Fig. 3D). Tumor angiogenesis can provide nutrients for tumor growth and promote malignant progression. Therefore, we examined the angiogenic ability of tumor cells after Mortalin knockdown by western blot assay. The results showed that Mortalin knockdown could inhibit the expression of VEGF protein in PC3 and DU145 cells. Meanwhile, in the angiogenesis assay, Mortalin knockdown significantly inhibited the ability of PC3 and DU145 cells to form blood vessels compared to the si-con group (Fig. 3E). Taken together, knockdown of Mortalin could inhibit the malignant progression of prostate cancer.

### Mortalin promotes EMT progression in prostate cancer

**Fig. 4:**
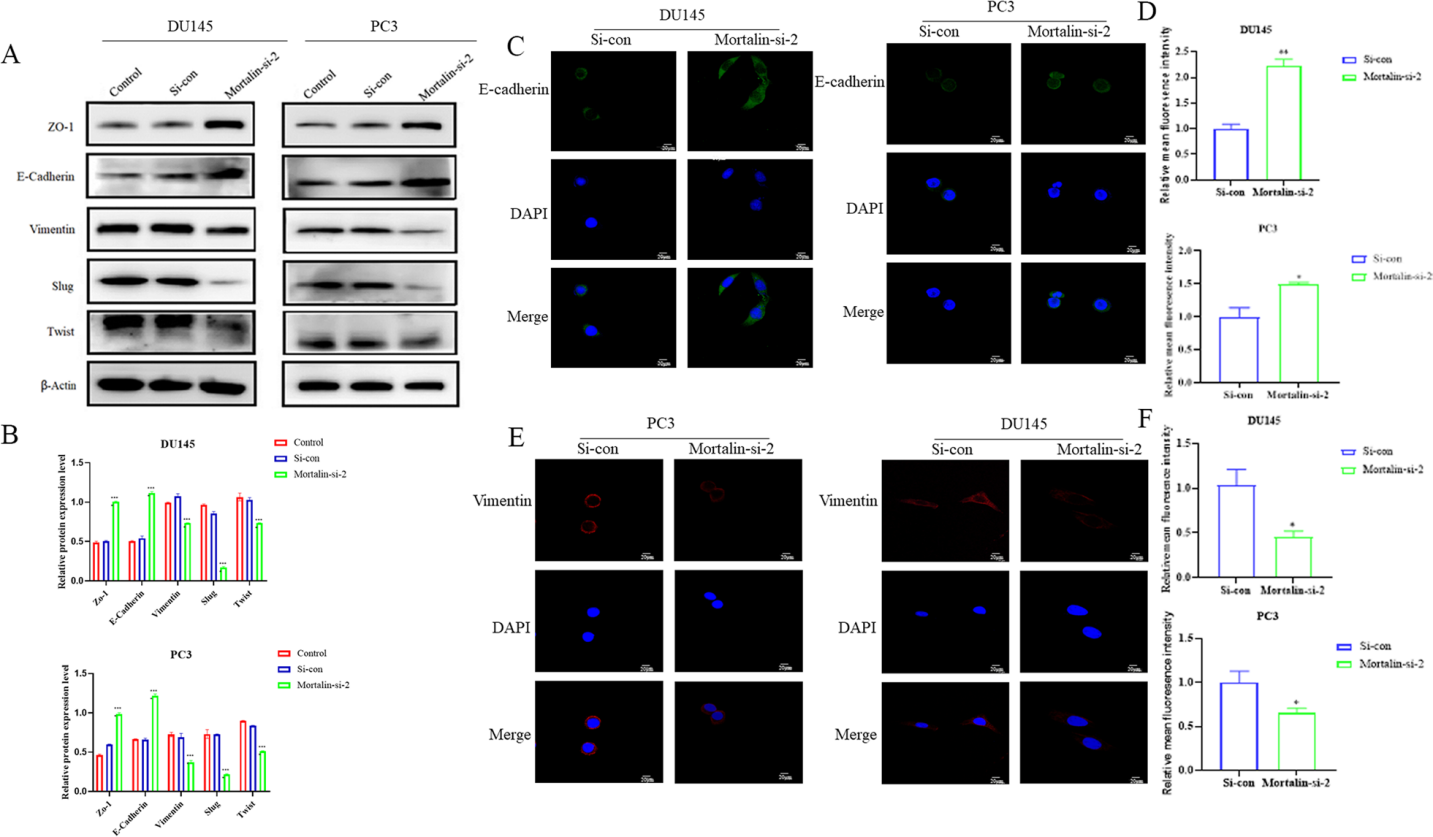
The relationship between Mortalin differential expression and tumor EMT. **A**, **B** Western blot was used to detect the expression differences of epithelial and mesenchymal markers in DU145 and PC3 cells, and western blotting histogram. **C** Immunofluorescence detection of difference in fluorescence signal intensity of E-cadherin between DU145 and PC3 cells. **D** Statistical histogram of group C data. **E** Immunofluorescence detection of difference in fluorescence signal intensity of Vimentin between DU145 and PC3 cells. **F** Statistical histogram of group E data. *P<0.05, **P<0.001   ***P < 0.001 VS Control

EMT is also called the transition of tumor epithelial cells to mesenchymal cells. The acceleration of EMT process can reduce the connection between cells and increase the possibility of tumor cell metastasis [21]. Therefore, the expression of protein markers of EMT in prostate cancer cells after Mortalin knockdown was detected by western blot (Fig. 4A, B). The results showed that after Mortalin knockdown, the expression of epithelial cell markers ZO-1 and E-Cadherin increased in PC3 and DU145 cells, while the expression of mesenchymal markers Vimentin, Slug and Twist decreased. Repeated verification by IF staining also demonstrated that in PC3 and DU145 cells, the expression of E-Cadherin was up-regulated and the expression of Vimentin was down-regulated after Mortalin knockdown (Fig. 4C–F; Additional file 1B-C).

### Mortalin promotes progression of the prostate cancer through the Wnt/β-catenin signaling pathway

**Fig. 5:**
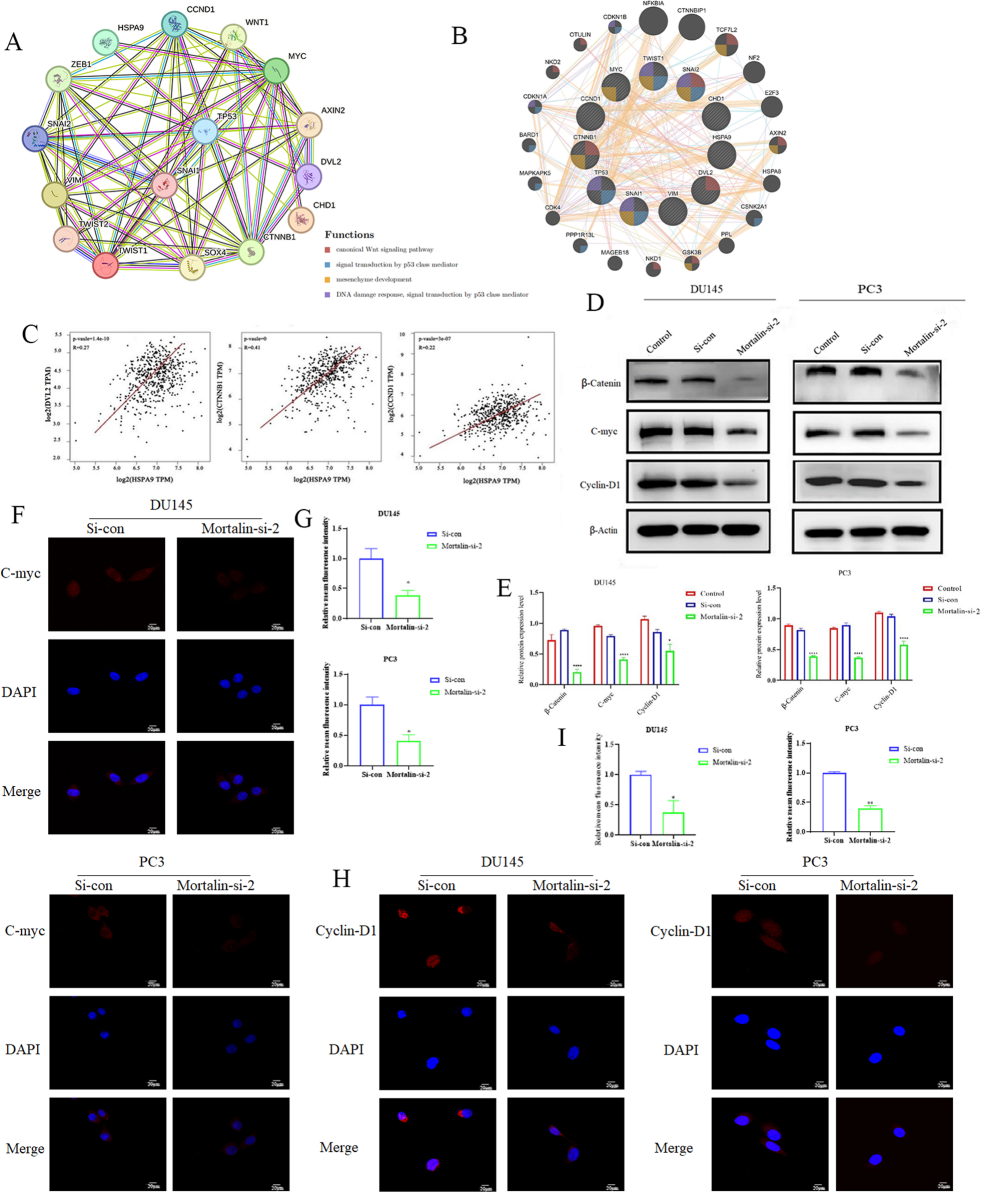
Wnt/β-catenin signaling pathway was affected by differential expression of Mortalin. **A**, **B** Interaction network between Wnt/β-Catenin signaling and EMT search by STRING and GeneMANIA database. **C** GEPIA database was used to detect the relationship in Mortalin, Wnt/β -catenin and Cyclin-D1 in prostate cancer. **D**, **E** Differential expression of Mortalin had effects on expression of β-catenin, C-myc and Cyclin-D1, and western blotting histogram. **F** Immunofluorescence detection of difference in fluorescence signal intensity of C-myc between DU145 and PC3 cells. **G** Statistical histogram of group F data. **H** Immunofluorescence detection of difference in fluorescence signal intensity of Cyclin-D1 between DU145 and PC3 cells. **I** Statistical histogram of group H data. * *P* < 0.05, **P<0.001 ****P < 0.0001 VS Control

Mortalin can be involved in tumor EMT process by activating Wnt/β-catenin signaling pathway. The Wnt/β-catenin signaling pathway plays an important role in cell cycle regulation and affects cell proliferation, differentiation and growth. It can lead to the accumulation of β-catenin in the nucleus, which interacts with transcription factors to promote the expression of target genes such as Wnt and cyclin D1 [22]. To verify this conclusion, We used STRING and GeneMANIA database analyses to demonstrate that the Wnt/β-catenin signaling pathway is correlated with EMT progression in prostate cancer first (Fig. 5A, B). Then, the analysis of GEPIA database showed that Mortalin was positively correlated with DVL2 (Wnt), CTNNB1 (β-catenin) and CCND1 (CyclinD1) in prostate cancer (Fig. 5C). Subsequently, Expression of proteins related to Wnt/β-catenin signaling pathway in DU145 and PC3 cells by Western blotting and immunofluorescence assay (Fig. 5F–I). The results showed that Mortalin knockdown significantly inhibited the expression of β-catenin, C-myc and Cyclin-D1. These results suggest that Mortalin knockdown can inhibit EMT process through Wnt/β-catenin signaling pathway and thus inhibit the malignant progression of prostate cancer. Single factor analysis showed that Gleason score, TNM stage, lymph node metastasis and Mortalin expression were closely correlated with poor prognosis of prostate cancer patients (Table 2). Multivariate analysis showed that lymph node metastasis and Mortalin expression were independent risk factors for poor prognosis in prostate cancer patients (Table 3). These results suggest that high Mortalin protein expression is an independent risk factor for poor prognosis in prostate cancer patients.

**Table 2:** Correlation analysis of Mortalin protein expression and clinicopathological parameters of prostate cancer patients

Variables	No. of cases	Strongly positive Mortalin cases +++ (%)	Weakly Mortalin cases −~++ (%)	χ^2^	*p* value
Age (years)				1.479	0.224
< 60	42	22 (52.4)	20 (47.6)		
≥ 60	48	19 (39.6)	29 (61.4)		
Tumor size				3.269	0.071
T1–T2	51	19 (37.3)	32 (62.7)		
T3–T4	39	22 (56.4)	17 (44.6)		
Gleason score				5.443	0.020*
≤ 6	36	11 (30.6)	25 (69.4)		
≥ 7	54	30 (55.6)	24 (44.4)		
TNM stage				6.868	0.009**
I–II	57	20 (35.1)	37 (64.9)		
III–IV	33	21 (63.6)	12 (36.4)		
LN metastasis				17.203	0.000***
Negative	54	15 (27.8)	39 (72.2)		
Positive	36	26 (72.2)	10 (27.8)		

^*^P < 0.05, **P < 0.01, ***P < 0.001

**Table 3:** Univariate and multivariate analysis of influencing prognosis of prostate cancer patients

Factors	B	SE	Wald	HR	95%CI		*p* value
					Lower	Upper	
Univariate survival analyses							
Age	0.023	0.289	0.006	1.023	0.580	1.804	0.937
Tumor size	0.001	0.294	0.000	1.001	0.563	1.780	0.997
Gleason score	0.705	0.344	4.194	2.023	1.031	3.971	0.041*
TNM stage	1.025	0.293	12.276	2.788	1.571	4.948	0.000***
LN metastasis	1.130	0.302	14.052	3.096	1.715	5.591	0.000***
Mortalin expression	1.441	0.336	18.400	4.225	2.187	8.162	0.000***
Multivariate survival analyses							
Gleason score	0.455	0.364	1.568	1.577	0.773	3.215	0.210
TNM stage	0.565	0.317	3.177	1.759	0.945	3.272	0.075
LN metastasis	0.676	0.341	3.919	1.965	1.007	3.836	0.048*
Mortalin expression	0.839	0.400	4.391	2.313	1.056	5.068	0.036*

*P < 0.05, ***P < 0.001

### Mortalin promotes prostate cancer progression in-vivo

**Fig. 6:**
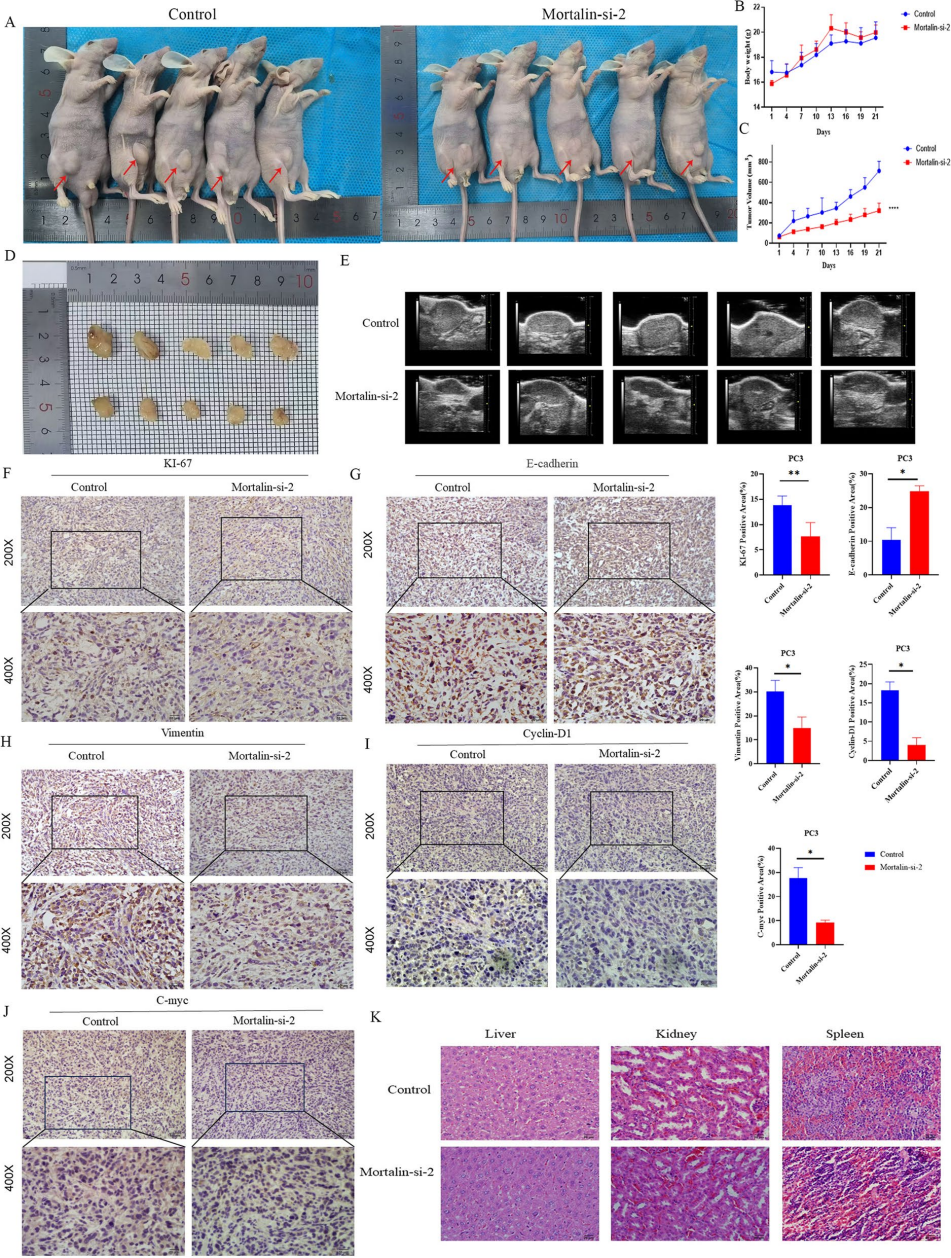
Differential expression of Mortalin inhibits tumor growth in-vivo. **A** Mortalin-si-2 inhibits tumor growth in-vivo. **B** Body weight of mice. **C** Tumors size and volume of mice; **D** Tumor growth was monitored for 21 days. After 21 days of treatment, tumors were removed and photographed. **E** Representative US images of Mortalin differential expression in PC3 tumors. **F**–**J** Expression of ki-67, E-cadherin, vimentin, Cyclin-D1 and C-myc in tumors tissues after subcutaneous injection detected by IHC. **K** H&E of liver, kidney, and spleen tissues isolated from each mouse group. * *P*<0.05, **P<0.001,  ****P < 0.0001 VS Control

To evaluate the effect of Mortalin on cancer metastasis, xenograft models in-vivo was applied. Intraperitoneally injections of PC3 cells knockdown Mortalin (Mortalin-si-2) or negative control (si-con) into BALB/c nude mice were carried out. The results showed that the tumor volume was reduced in the Mortalin-si-2 group compared with the si-con group. The Mortalin-si-2 group did not cause significant differences in the body weights of mice and the damage of normal tissues, and the absence of physical distress over the course of the experiment. (Fig. 6A–D). The Vevo2100 LAZR high frequency US imaging system demonstrated that Mortalin-si-2 group can reduce tumor formation (Fig. 6E). These findings strongly suggest that Mortalin significantly promotes cancer progression within an *in-vivo* model. The expression of Ki-67 (Fig. 6F), a marker associated with cellular proliferation, was significantly reduced following Mortalin-si-2 group. Consistent with *in-vitr*o findings, Immunohistochemical analysis revealed compelling evidence that the administration of Mortalin-si-2 group effectively suppressed the process of EMT transformation (Fig. 6G, H), Meanwhile, immunohistochemistry was performed to detect the expression of Cyclin-D1 and C-myc, the tumor cycle-related proteins in nude mice (Fig. 6I, J). Notably, HE staining demonstrated no discernible impact on the liver, kidney, and spleen, indicating the absence of adverse effects on these vital organs (Fig. 6k).

## Discussion

Prostate cancer is a major global health concern that primarily affects older individuals. The latest global cancer data for 2023 underscores its prominence as one of the most common cancers among men, with a substantial burden in terms of new morbidity and mortality [23]. Currently, androgen deprivation therapy (ADT) is mainly used to treat early-stage prostate cancer, but many patients will develop drug resistance and progress to androgen-independent prostate cancer when repeated therapy is used for a long time [24]. There are many factors affecting the occurrence of prostate cancer. Studies have found that the over-expression of Mortalin can promote the malignant progression of prostate cancer. Mortalin is involved in the proper folding of newly formed proteins and the renaturation of damaged proteins. It is also involved in the transport of specific proteins to appropriate organelles or lysosomes and protecting proteins from degradation factors produced during cellular stress. In addition, Mortalin is also involved in the regulation of immune response and apoptosis, affecting the sensitivity of cells to androgen receptor (AR) [25–27].

Many studies have shown that Mortalin protein interacts through the relationships existed in the HSP40/HSP70/HSP90 chaperone mechanism in prostate cancer cells, which is consistent with the versatility of the chaperone mechanism [28–30]. These data further support the study of HSP40/HSP70 companion shafts as a basis for androgen-independent hormone replacement therapy for prostate cancer.

Differences in the expression of Mortalin in normal Tumor-surrounding tissue and tumor tissues have been improved. Data analysis and IHC analysis were conducted by CCLE and UALCAN databases in this paper. It was confirmed that Mortalin expression was higher in prostate cancer tissues. In addition, our IHC results showed that Mortalin was more expressed in tumor tissue with higher Gleason scores, confirming the importance of Mortalin in the malignant progression of prostate cancer. Previously, our research team has shown that Mortalin plays an important role in the malignant progression of breast cancer [31] and lung adenocarcinoma [16]. It has been shown that Mortalin can promote the malignant progression of ovarian cancer. In-vivo and in-vitro data show that Mortalin plays an important role in the progression of many cancers, which promotes the proliferation, migration, invasion, and EMT process of tumor cells. It also fully demonstrates that Mortalin has great potential as a predictor of malignant progression of cancer and an evaluation index of tumor survival. These common findings also suggest the possibility of Mortalin as an indicator of cancer malignancy and a potential therapeutic target for androgen-independent prostate cancer.

To explore Mortalin’s specific mechanisms for promoting malignant progression in prostate cancer. We screened for androgen-independent prostate cancer cells with higher levels of malignancy, DU145 and PC3, through Western blot, and conducted a series of Mortalin related experiments. To verify Mortalin’s role in cell proliferation, we employed MTT and colony formation experiments to show that knocking down Mortalin inhibits the proliferation in DU145 and PC3 cells.

Tumor metastasis is the main cause of death in patients. Androgen independent prostate cancer is more likely to develop malignant progression due to its insensitivity to hormone therapy [32, 33]. Therefore, it is very important to inhibit tumor metastasis effectively. The results of scratch test and transwell test showed that Mortalin expression was negatively correlated with scratch healing rate. These results suggest that Mortalin knockout can be an effective method to inhibit the migration and progression of androgen independent prostate cancer. Tumor angiogenesis is an important link in tumor survival and progression [34], in which VEGF plays a vital role, and the expression of VEGF was detected by Western blot test. The effect of Mortalin differential expression on angiogenesis was examined by angiogenesis assay. The results showed that the ability of forming blood vessels was reduced after Mortalin knockdown in prostate cancer cells.

EMT affects and promotes metastasis cascade, which is characterized by loss of cell adhesion and cell membrane polarity, enhancing cell motility and metastasis [35, 36]. Meanwhile, Mortalin is also involved in tumor EMT process. We detected the expression levels of EMT markers in DU145 and PC3 cells by Western blot and IF staining. The results showed that the expression of epithelial markers was up-regulated and mesenchymal markers down-regulated after Mortalin knockdown. These results suggest that Mortalin promotes the EMT process in prostate cancer.

The Wnt/β-catenin pathway plays a key role in embryonic development and adult tissue homeostasis. Blocking this pathway can inhibit angiogenesis in prostate cancer [37, 38]. Therefore, angiogenesis assay was used to detect the effect of differential expression of Mortalin on angiogenesis, and Western blot assay was used to detect the expression levels of related proteins β-catenin, C-Myc and CyclinD1. The results showed that the expression levels of β-catenin, C-myc and CyclinD1 were decreased after Mortalin knockdown. In conclusion, Mortalin knockout can effectively inhibit tumor malignant progression by inhibiting Wnt/β-catenin signaling pathway, thus playing an anti-tumor role.

The up-regulation of Mortalin helps to improve the stemness of cancer cells and promote the increase of stemness markers, thus showing high migration ability and weak response to various cancer chemotherapy drugs. Single factor analysis confirmed that TNM stage, lymph node metastasis and Mortalin expression were independent factors for poor prognosis in prostate cancer patients. In the multivariate analysis, lymph node metastasis and Mortalin expression were combined to influence the poor prognosis of prostate cancer. These results suggest that Mortalin expression is not only closely related to the degree of malignancy of prostate cancer, but also can be an independent risk factor affecting the survival of patients. Our study provides evidence for the role of Mortalin in the progression of prostate cancer and suggests its potential as a therapeutic target. Further research is needed to explore the feasibility and effectiveness of targeting Mortalin in the treatment of prostate cancer, particularly in the context of androgen-independent disease.

In conclusion, down-regulation of Mortalin has an inhibitory effect on the malignant progression of prostate cancer and exerts a tumour-suppressive effect through the Wnt/β-catenin signaling pathway. In vivo and in vitro experiments demonstrated that Mortalin downregulation significantly affected proliferation, migration and epithelial-mesenchymal transition (EMT) in androgen-independent prostate cancer. These findings confirm that Mortalin may be a promising target in the treatment of androgen-dependent prostate cancer (Additional file 1D).

### Supplementary Information

**Additional file 1.** Mortalin promotes the evolution of androgen-independent prostate cancer through Wnt/β-catenin signaling pathway.

## Data Availability

All data are available on Pubmed, Gene Expression Omnibus (GEO) and Web of Science.

## References

[1] Kocarnik JM, Compton K, Dean FE, Fu W, Gaw BL, Harvey JD, Henrikson HJ, Lu D, Pennini A, Xu R, Ababneh E, Abbasi-Kangevari M, Abbastabar H, Abd-Elsalam SM, Abdoli A, Abedi A, Abidi H, Abolhassani H, Adedeji IA, Adnani QES, Advani SM, Afzal MS, Aghaali M, Ahinkorah BO, Ahmad S, Ahmad T, Ahmadi A, Ahmadi S, Ahmed Rashid T, Ahmed Salih Y, Akalu GT, Aklilu A, Akram T, Akunna CJ, Al Hamad H, Alahdab F, Al-Aly Z, Ali S, Alimohamadi Y, Alipour V, Aljunid SM, Alkhayyat M, Almasi-Hashiani A, Almasri NA, Al-Maweri SAA, Almustanyir S, Alonso N, Alvis-Guzman N, Amu H, Anbesu EW, Ancuceanu R, Ansari F, Ansari-Moghaddam A, Antwi MH, Anvari D, Anyasodor AE, Aqeel M, Arabloo J, Arab-Zozani M, Aremu O, Ariffin H, Aripov T, Arshad M, Artaman A, Arulappan J, Asemi Z, Asghari Jafarabadi M, Ashraf T, Atorkey P, Aujayeb A, Ausloos M, Awedew AF, Ayala Quintanilla BP, Ayenew T, Azab MA, Azadnajafabad S, Azari Jafari A, Azarian G, Azzam AY, Badiye AD, Bahadory S, Baig AA, Baker JL, Balakrishnan S, Banach M, Bärnighausen TW, Barone-Adesi F, Barra F, Barrow A, Behzadifar M, Belgaumi UI, Bezabhe WMM, Bezabih YM, Bhagat DS, Bhagavathula AS, Bhardwaj N, Bhardwaj P, Bhaskar S, Bhattacharyya K, Bhojaraja VS, Bibi S, Bijani A, Biondi A, Bisignano C, Bjørge T, Bleyer A, Blyuss O, Bolarinwa OA, Bolla SR, Braithwaite D, Brar A, Brenner H, Bustamante-Teixeira MT, Butt NS, Butt ZA, Caetano dos Santos FL, Cao Y, Carreras G, Catalá-López F, Cembranel F, Cerin E, Cernigliaro A, Chakinala RC, Chattu SK, Chattu VK, Chaturvedi P, Chimed-Ochir O, Cho DY, Christopher DJ, Chu DT, Chung MT, Conde J, Cortés S, Cortesi PA, Costa VM, Cunha AR, Dadras O, Dagnew AB, Dahlawi SMA, Dai X, Dandona L, Dandona R, Darwesh AM, das Neves J, De le Hoz FP, Demis AB, Denova-Gutiérrez E, Dhamnetiya D, Dhimal ML, Dhimal M, Dianatinasab M, Diaz D, Djalalinia S, Do HP, Doaei S, Dorostkar F, Dos Santos Figueiredo FW, Driscoll TR, Ebrahimi H, Eftekharzadeh S, El Tantawi M, El-Abid H, Elbarazi I, Elhabashy HR, Elhadi M, El-Jaafary SI, Eshrati B, Eskandarieh S, Esmaeilzadeh F, Etemadi A, Ezzikouri S, Faisaluddin M, Faraon EJA, Fares J, Farzadfar F, Feroze AH, Ferrero S, Ferro Desideri L, Filip I, Fischer F, Fisher JL, Foroutan M, Fukumoto T, Gaal PA, Gad MM, Gadanya MA, Gallus S, Gaspar Fonseca M, Getachew Obsa A, Ghafourifard M, Ghashghaee A, Ghith N, Gholamalizadeh M, Gilani SA, Ginindza TG, Gizaw ATT, Glasbey JC, Golechha M, Goleij P, Gomez RS, Gopalani SV, Gorini G, Goudarzi H, Grosso G, Gubari MIM, Guerra MR, Guha A, Gunasekera DS, Gupta B, Gupta VB, Gupta VK, Gutiérrez RA, Hafezi-Nejad N, Haider MR, Haj-Mirzaian A, Halwani R, Hamadeh RR, Hameed S, Hamidi S, Hanif A, Haque S, Harlianto NI, Haro JM, Hasaballah AI, Hassanipour S, Hay RJ, Hay SI, Hayat K, Heidari G, Heidari M, Herrera-Serna BY, Herteliu C, Hezam K, Holla R, Hossain MM, Hossain MBH, Hosseini MS, Hosseini M, Hosseinzadeh M, Hostiuc M, Hostiuc S, Househ M, Hsairi M, Huang J, Hugo FN, Hussain R, Hussein NR, Hwang BF, Iavicoli I, Ibitoye SE, Ida F, Ikuta KS, Ilesanmi OS, Ilic IM, Ilic MD, Irham LM, Islam JY, Islam RM, Islam SMS, Ismail NE, Isola G, Iwagami M, Jacob L, Jain V, Jakovljevic MB, Javaheri T, Jayaram S, Jazayeri SB, Jha RP, Jonas JB, Joo T, Joseph N, Joukar F, Jürisson M, Kabir A, Kahrizi D, Kalankesh LR, Kalhor R, Kaliyadan F, Kalkonde Y, Kamath A, Kameran Al-Salihi N, Kandel H, Kapoor N, Karch A, Kasa AS, Katikireddi SV, Kauppila JH, Kavetskyy T, Kebede SA, Keshavarz P, Keykhaei M, Khader YS, Khalilov R, Khan G, Khan M, Khan MN, Khan MAB, Khang YH, Khater AM, Khayamzadeh M, Kim GR, Kim YJ, Kisa A, Kisa S, Kissimova-Skarbek K, Kopec JA, Koteeswaran R, Koul PA, Koulmane Laxminarayana SL, Koyanagi A, Kucuk Bicer B, Kugbey N, Kumar GA, Kumar N, Kumar N, Kurmi OP, Kutluk T, La Vecchia C, Lami FH, Landires I, Lauriola P, Lee SW, Lee SWH, Lee WC, Lee YH, Leigh J, Leong E, Li J, Li MC, Liu X, Loureiro JA, Lunevicius R, Magdy Abd El Razek M, Majeed A, Makki A, Male S, Malik AA, Mansournia MA, Martini S, Masoumi SZ, Mathur P, McKee M, Mehrotra R, Mendoza W, Menezes RG, Mengesha EW, Mesregah MK, Mestrovic T, Miao Jonasson J, Miazgowski B, Miazgowski T, Michalek IM, Miller TR, Mirzaei H, Mirzaei HR, Misra S, Mithra P, Moghadaszadeh M, Mohammad KA, Mohammad Y, Mohammadi M, Mohammadi SM, Mohammadian-Hafshejani A, Mohammed S, Moka N, Mokdad AH, Molokhia M, Monasta L, Moni MA, Moosavi MA, Moradi Y, Moraga P, Morgado-da-Costa J, Morrison SD, Mosapour A, Mubarik S, Mwanri L, Nagarajan AJ, Nagaraju SP, Nagata C, Naimzada MD, Nangia V, Naqvi AA, Narasimha Swamy S, Ndejjo R, Nduaguba SO, Negoi I, Negru SM, Neupane Kandel S, Nguyen CT, Nguyen HLT, Niazi RK, Nnaji CA, Noor NM, Nuñez-Samudio V, Nzoputam CI, Oancea B, Ochir C, Odukoya OO, Ogbo FA, Olagunju AT, Olakunde BO, Omar E, Omar Bali A, Omonisi AEE, Ong S, Onwujekwe OE, Orru H, Ortega-Altamirano DV, Otstavnov N, Otstavnov SS, Owolabi MO, Padubidri JR, Pakshir K, Pana A, Panagiotakos D, Panda-Jonas S, Pardhan S, Park EC, Park EK, Pashazadeh Kan F, Patel HK, Patel JR, Pati S, Pattanshetty SM, Paudel U, Pereira DM, Pereira RB, Perianayagam A, Pillay JD, Pirouzpanah S, Pishgar F, Podder I, Postma MJ, Pourjafar H, Prashant A, Preotescu L, Rabiee M, Rabiee N, Radfar A, Radhakrishnan RA, Radhakrishnan V, Rafiee A, Rahim F, Rahimzadeh S, Rahman M, Rahman MA, Rahmani AM, Rajai N, Rajesh A, Rakovac I, Ram P, Ramezanzadeh K, Ranabhat K, Ranasinghe P, Rao CR, Rao SJ, Rawassizadeh R, Razeghinia MS, Renzaho AMN, Rezaei N, Rezaei N, Rezapour A, Roberts TJ, Rodriguez JAB, Rohloff P, Romoli M, Ronfani L, Roshandel G, Rwegerera GM, Sabour S, Saddik B, Saeed U, Sahebkar A, Sahoo H, Salehi S, Salem MR, Salimzadeh H, Samaei M, Samy AM, Sanabria J, Sankararaman S, Santric-Milicevic MM, Sardiwalla Y, Sarveazad A, Sathian B, Sawhney M, Saylan M, Schneider IJC, Sekerija M, Seylani A, Shafaat O, Shaghaghi Z, Shaikh MA, Shamsoddin E, Shannawaz M, Sharma R, Sheikh A, Sheikhbahaei S, Shetty A, Shetty JK, Shetty PH, Shibuya K, Shirkoohi R, Shivakumar KM, Shivarov V, Siabani S, Siddappa Malleshappa SK, Silva DAS, Singh JA, Sintayehu Y, Skryabin VY, Skryabina AA, Soeberg MJ, Sofi-Mahmudi A, Sotoudeh H, Steiropoulos P, Straif K, Subedi R, Sufiyan MB, Sultan I, Sultana S, Sur D, Szerencsés V, Szócska M, Tabarés-Seisdedos R, Tabuchi T, Tadbiri H, Taherkhani A, Takahashi K, Talaat IM, Tan KK, Tat VY, Tedla BAA, Tefera YG, Tehrani-Banihashemi A, Temsah MH, Tesfay FH, Tessema GA, Thapar R, Thavamani A, Thoguluva Chandrasekar V, Thomas N, Tohidinik HR, Touvier M, Tovani-Palone MR, Traini E, Tran BX, Tran KB, Tran MTN, Tripathy JP, Tusa BS, Ullah I, Ullah S, Umapathi KK, Unnikrishnan B, Upadhyay E, Vacante M, Vaezi M, Valadan Tahbaz S, Velazquez DZ, Veroux M, Violante FS, Vlassov V, Vo B, Volovici V, Vu GT, Waheed Y, Wamai RG, Ward P, Wen YF, Westerman R, Winkler AS, Yadav L, Yahyazadeh Jabbari SH, Yang L, Yaya S, Yazie TSY, Yeshaw Y, Yonemoto N, Younis MZ, Yousefi Z, Yu C, Yuce D, Yunusa I, Zadnik V, Zare F, Zastrozhin MS, Zastrozhina A, Zhang J, Zhong C, Zhou L, Zhu C, Ziapour A, Zimmermann IR, Fitzmaurice C, Murray CJL, Force LM. Cancer incidence, mortality, years of life lost, years lived with disability, and disability-adjusted life years for 29 cancer groups from 2010 to 2019: a systematic analysis for the global burden of disease study 2019. JAMA Oncol. 2022; 8: 420–444. 10.1001/jamaoncol.2021.6987 PMC8719276 34967848

[2] Sung H, Ferlay J, Siegel RL, Laversanne M, Soerjomataram I, Jemal A, Bray F (2021). Global cancer statistics 2020: GLOBOCAN estimates of incidence and mortality worldwide for 36 cancers in 185 countries. CA Cancer J Clin.

[3] Siegel RL, Miller KD, Wagle NS, Jemal A (2023). Cancer statistics, 2023. CA Cancer J Clin.

[4] Druskin SC, Mamawala M, Tosoian JJ, Epstein JI, Pavlovich CP, Carter HB, Trock BJ (2019). Older age predicts biopsy and radical prostatectomy grade reclassification to aggressive prostate cancer in men on active surveillance. J Urol.

[5] Desai K, McManus JM, Sharifi N (2021). Hormonal therapy for prostate cancer. Endocr Rev.

[6] Tarique M, Naz H, Suhail M, Turan A, Saini C, Muhammad N, Shankar H, Zughaibi TA, Khan TH, Khanna N, Sharma A (2023). Differential expression of programmed death 1 (PD-1) on various immune cells and its role in human leprosy. Front Immunol.

[7] Trnski D, Sabol M, Tomić S, Štefanac I, Mrčela M, Musani V, Rinčić N, Kurtović M, Petrić T, Levanat S, Ozretić P (2021). SHH-N non-canonically sustains androgen receptor activity in androgen-independent prostate cancer cells. Sci Rep.

[8] Kvízová J, Pavlíčková V, Kmoníčková E, Ruml T, Rimpelová S (2021). Quo vadis advanced prostate cancer therapy? Novel treatment perspectives and possible future directions. Molecules.

[9] Benbrook DM (2022). SHetA2 attack on mortalin and colleagues in cancer therapy and prevention. Front Cell Dev Biol.

[10] Rai R, Kennedy AL, Isingizwe ZR, Javadian P, Benbrook DM (2021). Similarities and differences of Hsp70, hsc70, Grp78 and mortalin as cancer biomarkers and drug targets. Cells.

[11] Elwakeel A (2022). Abrogating the interaction between p53 and Mortalin (Grp75/HSPA9/mtHsp70) for cancer therapy: the story so far. Front Cell Dev Biol.

[12] Esfahanian N, Knoblich CD, Bowman GA, Rezvani K (2023). Mortalin: Protein partners, biological impacts, pathological roles, and therapeutic opportunities. Front Cell Dev Biol.

[13] Teng M, Hu C, Yang B, Xiao W, Zhou Q, Li Y, Li Z (2021). Salvianolic acid B targets mortalin and inhibits the migration and invasion of hepatocellular carcinoma via the RECK/STAT3 pathway. Cancer Cell Int.

[14] Huang X, Yan Y, Gui A, Zhu S, Qiu S, Chen F, Liu W, Zuo J, Yang L (2022). A regulatory loop involving miR-200c and NF-κB Modulates mortalin expression and increases cisplatin sensitivity in an ovarian cancer cell line model. Int J Mol Sci.

[15] Yoon AR, Wadhwa R, Kaul SC, Yun CO (2022). Why is mortalin a potential therapeutic target for cancer?. Front Cell Dev Biol.

[16] Meng Z, Zhang R, Wu X, Zhang M, Zhang S, Jin T (2022). Prognostic value of Mortalin correlates with roles in epithelial-mesenchymal transition and angiogenesis in lung adenocarcinoma. Carcinogenesis.

[17] Rajtak A, Czerwonka A, Pitter M, Kotarski J, Okła K (2023). Clinical relevance of mortalin in ovarian cancer patients. Cells.

[18] Brabletz S, Schuhwerk H, Brabletz T, Stemmler MP (2021). Dynamic EMT: a multi-tool for tumor progression. Embo j.

[19] Wei B, Cao J, Tian JH, Yu CY, Huang Q, Yu JJ, Ma R, Wang J, Xu F, Wang LB (2021). Mortalin maintains breast cancer stem cells stemness via activation of Wnt/GSK3β/β-catenin signaling pathway. Am J Cancer Res.

[20] Dai Y, Li F, Jiao Y, Wang G, Zhan T, Xia Y, Liu H, Yang H, Zhang J, Tang L (2021). Mortalin/glucose-regulated protein 75 promotes the cisplatin-resistance of gastric cancer via regulating anti-oxidation/apoptosis and metabolic reprogramming. Cell Death Discov.

[21] Yang J, Antin P, Berx G, Blanpain C, Brabletz T, Bronner M, Campbell K, Cano A, Casanova J, Christofori G, Dedhar S, Derynck R, Ford HL, Fuxe J, García de Herreros A, Goodall GJ, Hadjantonakis AK, Huang RYJ, Kalcheim C, Kalluri R, Kang Y, Khew-Goodall Y, Levine H, Liu J, Longmore GD, Mani SA, Massagué J, Mayor R, McClay D, Mostov KE, Newgreen DF, Nieto MA, Puisieux A, Runyan R, Savagner P, Stanger B, Stemmler MP, Takahashi Y, Takeichi M, Theveneau E, Thiery JP, Thompson EW, Weinberg RA, Williams ED, Xing J, Zhou BP, Sheng G (2020). Guidelines and definitions for research on epithelial-mesenchymal transition. Nat Rev Mol Cell Biol.

[22] Losada-García A, Salido-Guadarrama I, Cortes-Ramirez SA, Cruz-Burgos M, Morales-Pacheco M, Vazquez-Santillan K, Rodriguez-Martinez G, González-Ramírez I, Gonzalez-Covarrubias V, Perez-Plascencia C, Rodríguez-Dorantes M (2023). SFRP1 induces a stem cell phenotype in prostate cancer cells. Front Cell Dev Biol.

[23] Rebello RJ, Oing C, Knudsen KE, Loeb S, Johnson DC, Reiter RE, Gillessen S, Van der Kwast T, Bristow RG (2021). Prostate cancer. Nat Rev Dis Primers.

[24] Yanagisawa T, Rajwa P, Thibault C, Gandaglia G, Mori K, Kawada T, Fukuokaya W, Shim SR, Mostafaei H, Motlagh RS, Quhal F, Laukhtina E, Pallauf M, Pradere B, Kimura T, Egawa S, Shariat SF (2022). Androgen receptor signaling inhibitors in addition to docetaxel with androgen deprivation therapy for metastatic hormone-sensitive prostate cancer: a systematic review and meta-analysis. Eur Urol.

[25] Nunzio CDE, Fiori C, Fusco F, Gregori A, Pagliarulo V, Alongi F (2022). Androgen deprivation therapy and cardiovascular risk in prostate cancer. Minerva Urol Nephrol.

[26] Luo J, Wang D, Wan X, Xu Y, Lu Y, Kong Z, Li D, Gu W, Wang C, Li Y, Ji C, Gu S, Xu Y (2020). Crosstalk between AR and Wnt signaling promotes castration-resistant prostate cancer growth. Onco Targets Ther.

[27] Ratajczak W, Lubkowski M, Lubkowska A (2022). Heat shock proteins in benign prostatic hyperplasia and prostate cancer. Int J Mol Sci.

[28] Hoter A, Rizk S, Naim HY (2019). The multiple roles and therapeutic potential of molecular chaperones in prostate cancer. Cancers (Basel)..

[29] Kabakov AE, Gabai VL (2021). HSP70s in breast cancer: promoters of tumorigenesis and potential targets/tools for therapy. Cells.

[30] Rice MA, Kumar V, Tailor D, Garcia-Marques FJ, Hsu EC, Liu S, Bermudez A, Kanchustambham V, Shankar V, Inde Z, Alabi BR, Muruganantham A, Shen M, Pandrala M, Nolley R, Aslan M, Ghoochani A, Agarwal A, Buckup M, Kumar M, Going CC, Peehl DM, Dixon SJ, Zare RN, Brooks JD, Pitteri SJ, Malhotra SV, Stoyanova T (2022). SU086, an inhibitor of HSP90, impairs glycolysis and represents a treatment strategy for advanced prostate cancer. Cell Rep Med.

[31] Zhang R, Meng Z, Wu X, Zhang M, Zhang S, Jin T (2021). Mortalin promotes breast cancer malignancy. Exp Mol Pathol.

[32] Cheng Q, Butler W, Zhou Y, Zhang H, Tang L, Perkinson K, Chen X, Jiang XS, McCall SJ, Inman BA, Huang J (2022). Pre-existing castration-resistant prostate cancer-like cells in primary prostate cancer promote resistance to hormonal therapy. Eur Urol.

[33] Rudzinski JK, Govindasamy NP, Lewis JD, Jurasz P (2020). The role of the androgen receptor in prostate cancer-induced platelet aggregation and platelet-induced invasion. J Thromb Haemost.

[34] Kabir AU, Subramanian M, Lee DH, Wang X, Krchma K, Wu J, Naismith T, Halabi CM, Kim JY, Pulous FE, Petrich BG, Kim S, Park HC, Hanson PI, Pan H, Wickline SA, Fremont DH, Park C, Choi K (2021). Dual role of endothelial Myct1 in tumor angiogenesis and tumor immunity. Sci Transl Med.

[35] Bakir B, Chiarella AM, Pitarresi JR, Rustgi AK (2020). EMT, MET, plasticity, and tumor metastasis. Trends Cell Biol.

[36] Nowak E, Bednarek I (2021). Aspects of the epigenetic regulation of EMT related to cancer metastasis. Cells.

[37] Koirala S, Klein J, Zheng Y, Glenn NO, Eisemann T, Fon Tacer K, Miller DJ, Kulak O, Lu M, Finkelstein DB, Neale G, Tillman H, Vogel P, Strand DW, Lum L, Brautigam CA, Pascal JM, Clements WK, Potts PR (2020). Tissue-specific regulation of the Wnt/β-catenin pathway by PAGE4 inhibition of tankyrase. Cell Rep.

[38] Murillo-Garzón V, Kypta R (2017). WNT signalling in prostate cancer. Nat Rev Urol.

