# Supplementary figures and images for: Mortalin promotes the evolution of androgen-independent prostate cancer through Wnt/β-catenin signaling pathway
Source: Cancer Cell Int. 2024 Jun 7;24:203. doi: 10.1186/s12935-024-03345-x (PMC11162088; doi:10.1186/s12935-024-03345-x)

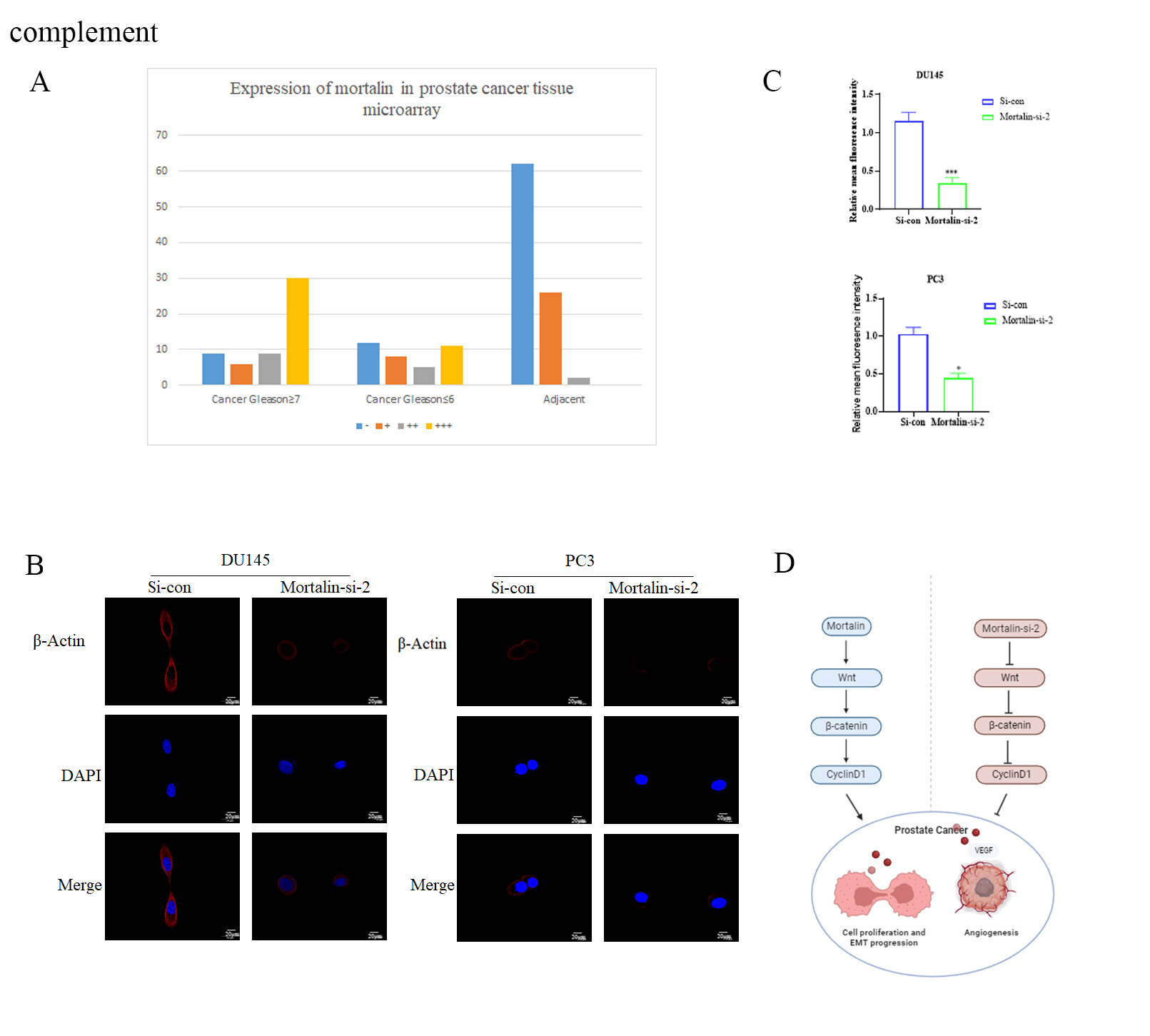

Supplement: Supplementary file 1 — Additional file 1. Mortalin promotes the evolution of androgen-independent prostate cancer through Wnt/β-catenin signaling pathway. [file 12935_2024_3345_MOESM1_ESM.png]
